# Multipurpose monitoring system for edible insect breeding based on machine learning

**DOI:** 10.1038/s41598-022-11794-5

**Published:** 2022-05-12

**Authors:** Paweł Majewski, Piotr Zapotoczny, Piotr Lampa, Robert Burduk, Jacek Reiner

**Affiliations:** 1 Faculty of Information and Communication Technology, Wrocław University of Science and Technology, Wrocław, Poland; 2 Department of Systems Engineering, University of Warmia and Mazury in Olsztyn, Olsztyn, Poland; 3 Faculty of Mechanical Engineering, Wrocław University of Science and Technology, Wrocław, Poland

**Keywords:** Mechanical engineering, Computational science, Computer science, Scientific data, Software

## Abstract

The Tenebrio molitor has become the first insect added to the catalogue of novel foods by the European Food Safety Authority due to its rich nutritional value and the low carbon footprint produced during its breeding. The large scale of Tenebrio molitor breeding makes automation of the process, which is supported by a monitoring system, essential. Present research involves the development of a 3-module system for monitoring Tenebrio molitor breeding. The instance segmentation module (ISM) detected individual growth stages (larvae, pupae, beetles) of Tenebrio molitor, and also identified anomalies: dead larvae and pests. The semantic segmentation module (SSM) extracted feed, chitin, and frass from the obtained image. The larvae phenotyping module (LPM) calculated features for both individual larvae (length, curvature, mass, division into segments, and their classification) and the whole population (length distribution). The modules were developed using machine learning models (Mask R-CNN, U-Net, LDA), and were validated on different samples of real data. Synthetic image generation using a collection of labelled objects was used, which significantly reduced the development time of the models and reduced the problems of dense scenes and the imbalance of the considered classes. The obtained results (average $F1>0.88$ for ISM and average $F1>0.95$ for SSM) confirm the great potential of the proposed system.

## Introduction

The current problems of feeding an ever-increasing human population involve meeting the demand for animal protein without the environmental costs associated with animal husbandry. Preference is given to livestock systems that use less water, minimise space and reduce greenhouse gas emissions. The United Nations (UN) predicts that human protein consumption will reach 39 grams per day in 2030, and 57 grams in 2050^1^. The solution to this problem may be industrial insect breeding with minimised human labour and high stocking rates per unit building area. This is important, because in recent years, according to the recommendations of good husbandry practices, there has been an aim to reduce the stocking density per 1$\hbox {m}^2$ of building area of the main livestock species such as cattle, pigs and poultry. Moreover, we have also observed huge problems with African swine fever (ASF) and Avian influenza (AI), causing many livestock buildings to close with no idea of how to then use them. One alternative for their reuse could be intensive breeding of insects for food and feed^2^. According to the International Platform of Insects for Food and Feed (IPIFF), within the next 10 years the insect sector will become an integral part of the European agri-food chain. It is forecast that 1 in 10 fish consumed in the European Union (EU) will come from fish farms that use insect protein in their feed, 1 in 4 eggs consumed in Europe will come from insect-fed laying hens, 1 in 5 servings of chicken meat will come from insect-fed broilers, and 1 in 100 servings of pork will come from insect-fed pigs.

Insects are the most numerous group of known animal species, and they are the most important element of the ecosystem^2^. They are a valuable source of protein for many animal species and for people living in Africa, Asia or South America. Of the millions of insect species, more than 2000 are recognised as being edible. In Europe, there is no tradition of eating insects as a protein substitute. For now, most are bred only as protein and fat supplements to feed other animals. This is possible because the EU has approved insect protein for the production of feed for fish, poultry and pigs. Additionally, in 2021, after many studies, the European Food Safety Authority showed that Tenebrio molitor larvae are a rich source of protein, fat and fiber, and included it in the catalogue of novel foods. Thus, whole or powdered dried larvae can be an ingredient in pasta, cookies, and other food products. However, for such breeding to be profitable, industrial production technologies for selected insect species, such as Tenebrio molitor, must be developed in order to provide a standardised and cost-competitive product to the market.

Tenebrio molitor is a beetle from the Darkling beetles (Tenebrionidae) family. Adults have an elongated body measuring 12–18 mm in length. In the life cycle of Tenebrio molitor, the following can be distinguished: eggs, larvae, pupae and beetles. The larvae transform into a pupae in 45–60 days after 7–9 moult cycles, and the pupae transform into beetles after 5 next days^3^.

Industrial livestock production technology involves fully automating almost all animal handling operations through robots supervised by vision systems and a set of sensors associated with a production management module. If industrial breeding of Tenebrio molitor is to be profitable, individual breeding processes should also be automated. Its breeding systems currently involve keeping a certain number of larvae in boxes that are stacked on racks at several or more levels. Most of the work performed is manual and labour intensive. Today, with the exception of a few very large operators that are capable of producing several thousand tons of insects, the vast majority of insect farms are startups and small or medium-sized companies with little or no automation in their production systems. Keeping in mind the recommendation for breeding^4^, the activities in the production of Tenebrio molitor are: (i) feeding, (ii) wetting of larvae, (iii) sorting of larvae into size classes, (iv) harvesting of chitinous moult, (v) final harvesting of larvae and the separation of them from impurities. In order to control the ongoing effects of breeding, it is necessary to measure: (1) biomass gains, (2) the amount of chitinous moult, (3) the amount of dead larvae, (4) the amount of consumed feed, (5) the amount of possible pests (Alphitobius diaperinus), and (6) the number of individuals after transformation to pupae or beetles. In view of the requirements, fully automating production is not something that is easy. The basis of farm automation can be a vision system based on RGB cameras, or cameras outside the visible range (UV, IR). However, the problem is not with the hardware, but with the software. While the availability of cameras is very high, there is a lack of information in literature on the algorithms that can identify even the basic parameters of Tenebrio molitor breeding. This problem is difficult to solve because the objects to be identified overlap, and the colour or texture of each instance is very similar to each other.

Image analysis methods have more and more applications in precision agriculture. They are commonly used to assess the quality of raw materials and food products^5^. There is also research using vision systems and soil worms to assess drug effects. Digital fluorescence images of Caenorhabditis elegans worms were captured with a CCD camera, and the lymph flow through the worms’ bodies was determined based on the developed algorithms (Migliozzi et al., 2019)^6^. Tao et al.^7^ presented the results of identifying the sex of silkworm pupae using vision systems based on hyperspectral cameras. They used the successive projections algorithm (SPA) for variable selection^8^, gray-level co-occurrence matrix (GLCM)^9^analysis, and support vector machines (SVM)^10^ and radial basis function neural network (RBF-NN) models to achieve more than 98% accuracy in identifying the sex of silkworm pupae. Similar results were obtained by Sumriddetchkajorn et al.^11^ except that they obtained images of silkworm pupae by illuminating the cocoons with light from diodes and then capturing the images with a CCD camera. A combination of near-infrared hyperspectral imaging, convolutional neural networks (CNN), and a capsule network^12^ allowed for the identification of the storage pest (khapra beetle, Trogoderma granarium Everts) with over 90 percent accuracy (Agarwal et al.)^13^. A study on determining the developmental stage of pupals using vision systems was conducted by Sasha et al.^14^ for two species of blowfly (Diptera: Calliphoridae).

Unfortunately, there are only a few articles on the use of image analysis to automate Tenebrio molitor production. Companies with solutions, such as Dilepix, offer off-the-shelf systems, but do not provide details of their solutions. Kröncke et al.^15^ presented a system for automating the production of Tenebrio molitor. They developed a pneumatic system for separating larvae from impurities. They also proposed a method to evaluate the health and developmental status of larvae using a vision system. For this purpose, they classified image fragments into three classes: good segments, bad segments, and artifacts with the use of a multi-layer perceptron neural network (MLP-NN), which achieved an accuracy of 95.4%.

Due to a lack of sufficient knowledge on the development of complex systems for the automatic production and industrial control of Tenebrio molitor, the authors undertook to develop such a system. Its key element is a vision system, the tasks of which include the automatic identification of individual instances and the calculation of production parameters, which are the basis for the control of the entire farm. The main achievements of our research are: (1) a multipurpose monitoring system for edible insect breeding based on machine learning, (2) a novel non-invasive method for calculating the mass of Tenebrio molitor larvae based on images, (3) a novel method for estimating the size distribution of objects in dense scenes, (4) an original method for developing models for multiclass instance and semantic segmentation based on synthetic image generation and a partially automated labelling process.

## Methods

This chapter describes the successive steps of the iterative development of machine learning models, from defining problems and system concept to the evaluation of the proposed methods.

### Problem definition and system concept

**Figure 1:**
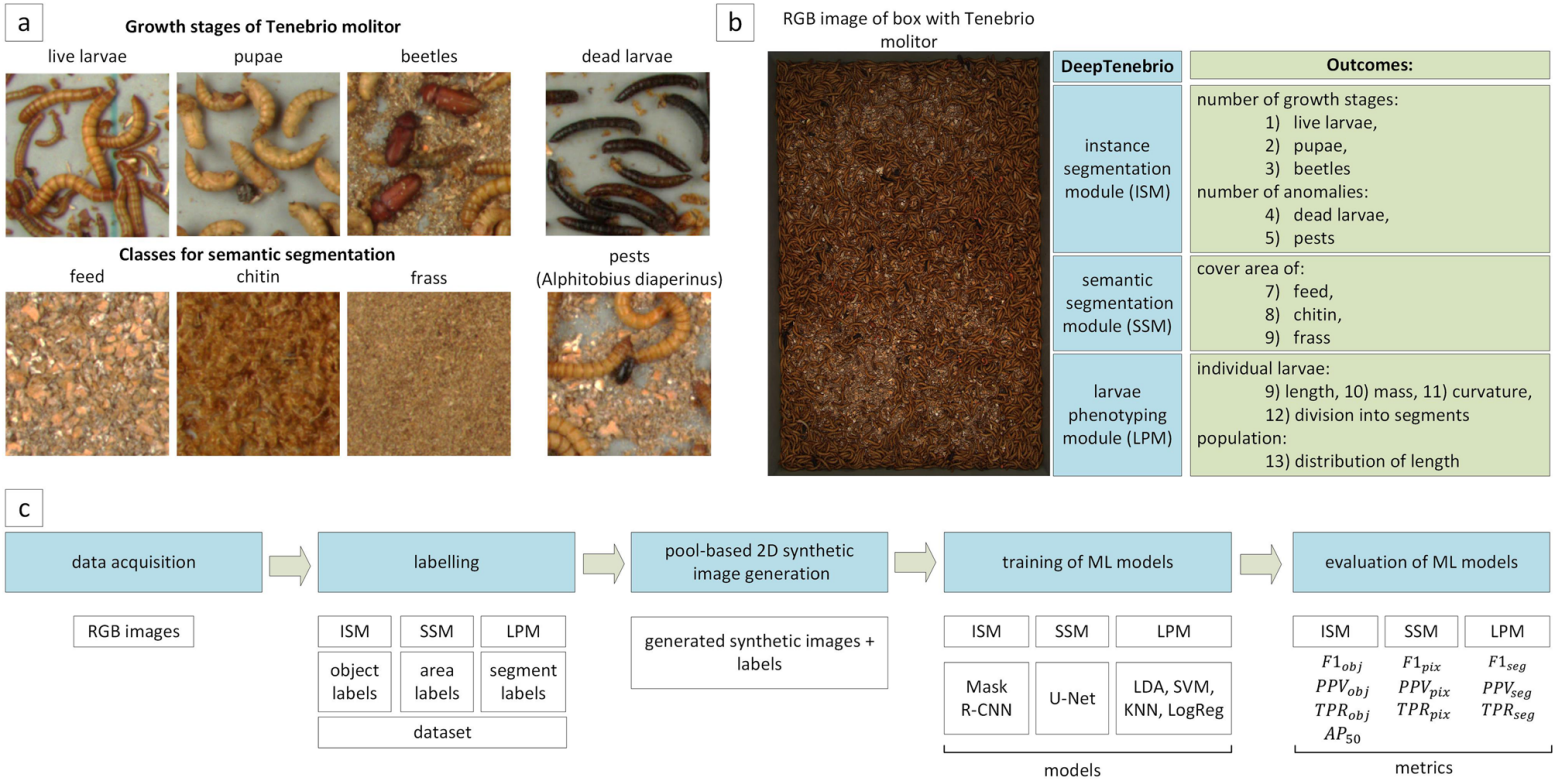
Presentation of the addressed problem: (**a**) the defined object classes used in the study, (**b**) the concept of the proposed 3-module DeepTenebrio system, (**c**) the next steps in developing the machine learning models for the proposed modules.

We divided the addressed problems into 3 groups: those requiring instance segmentation, those requiring semantic segmentation, or those related to larvae phenotyping. The first group involved the detection and segmentation of the growth stages of Tenebrio molitor (live larvae, pupae, beetles), and also anomalies in the form of dead larvae and pests (Alphitobius diaperinus). The instance segmentation module (ISM) determined the number of objects belonging to each class, their location, and an extracted binary mask for further phenotyping of individual instances. The second semantic segmentation module (SSM) extracted areas that represent the densities of the feed, chitin and frass from the image, and then calculated the percentage of these areas in the whole image. The third module was related to larvae phenotyping (LPM) at the level of both individual larvae (calculation of length, curvature, mass, division into segments, and their classification) and the whole population (determination of length distribution). The defined object classes used in the study (a) and the concept of the proposed 3-module DeepTenebrio system (b) are shown in Fig. 1.

Figure 1 also shows the next steps in developing the machine learning models for the proposed modules (c), which are characterised in the following sections.

### Data acquisition

**Figure 2:**
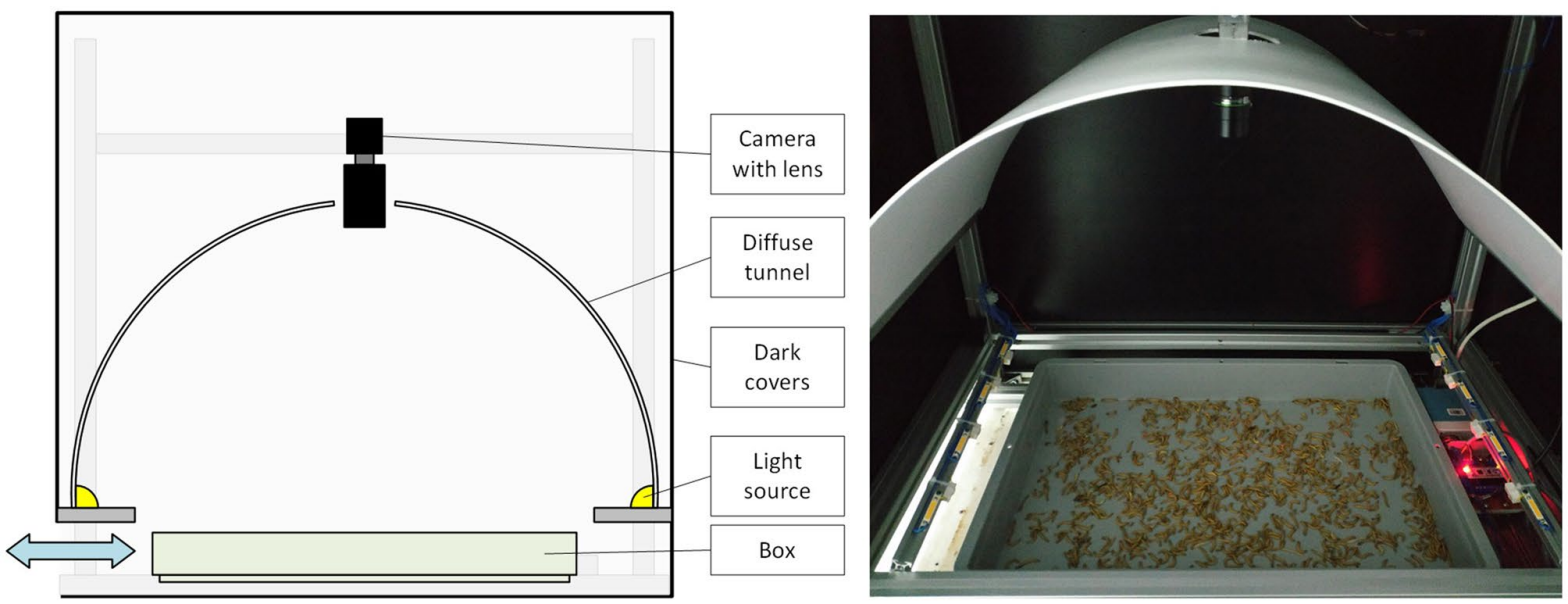
Data acquisition station.

The place for breeding Tenebrio molitor were boxes placed on the shelves of racks. Selected boxes with Tenebrio molitor were taken off the shelves and put into the data acquisition station. Its schematic diagram, with a real photo, is shown in Fig. 2.

The station allowed high-resolution RGB images in manual and automatic modes to be collected. The Phoenix PHX120S-CC camera (LUCID Vision Labs, Canada) with a resolution of 4096 x 3000 pixels was selected for image acquisition. The boxes with Tenebrio molitor were illuminated with a neutral white light (colour temperature 4000K) that was scattered in a diffuse tunnel. To reduce insect stress, the illuminators were only triggered for a short time for the duration of camera exposure. The covers isolated the image acquisition area from external factors. In total, 120 raw images of boxes with Tenebrio Molitor under breeding conditions were collected as a basis for labeling and developing the proposed modules. The selected populations in boxes differed in the growth stage of individuals, presence of anomalies, amount of uneaten food and chitin. Data were collected at Tenebria (Lubawa, Poland).

### Labelling

Labelling is an integral part of developing machine learning models, and allows the transfer of annotator knowledge to the model through its supervised training. This section describes the adopted data labelling strategy for training the models for the following modules: ISM, SSM, and LPM, as shown in Fig. 1.

#### First stage of labelling

**Figure 3:**
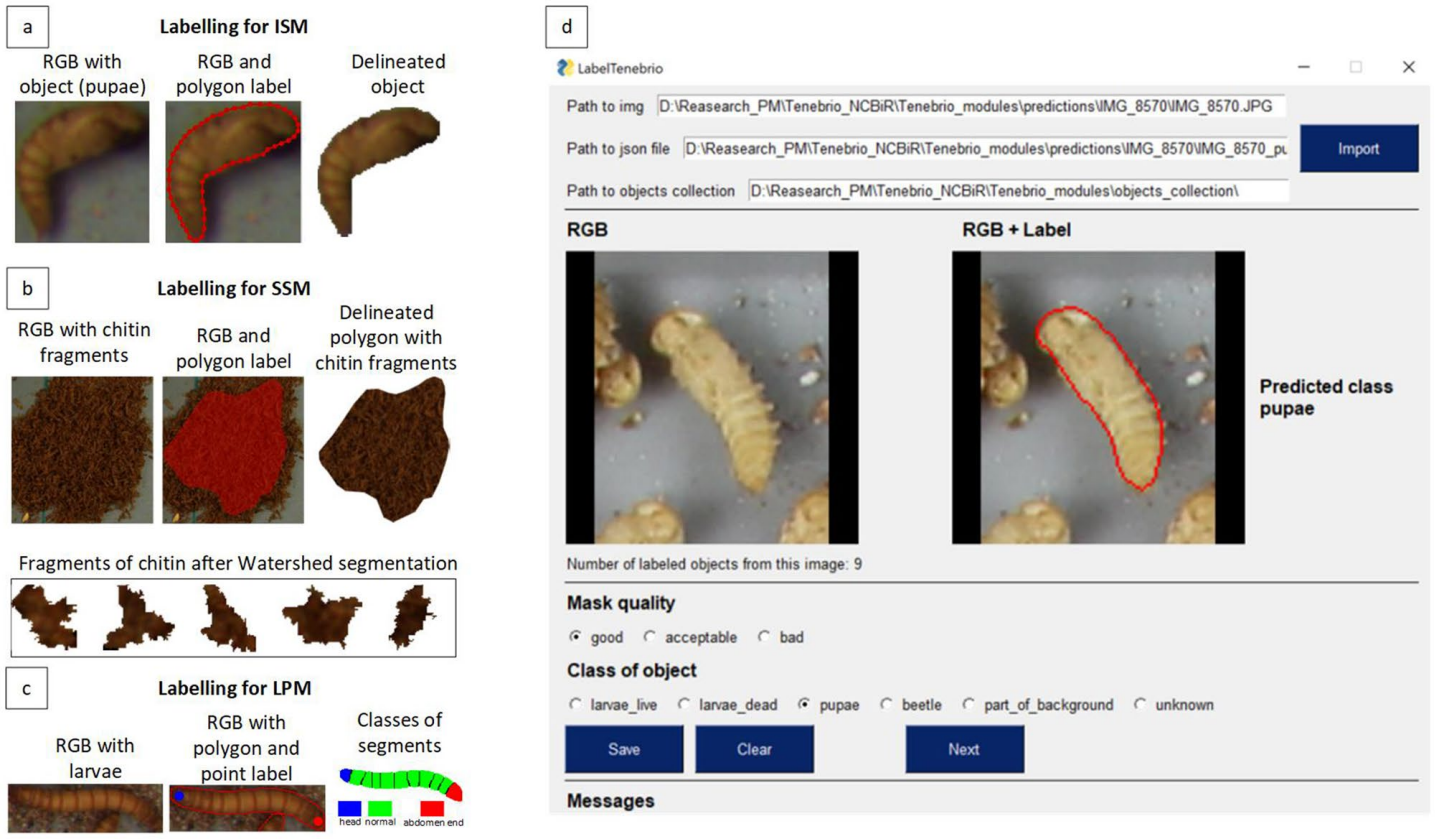
Methods and tools used for the labelling: (**a**) object labelling for the ISM, (**b**) area labelling for the SSM, (**c**) larva segment labelling for the LPM, (**d**) LabelTenebrioApp for improving the instance labelling process.

The first stage of labelling consisted of manual annotation of the images. For each proposed module: ISM, SSM, and LPM, the forms of annotation were different, and are shown in Fig. 3.

In the case of the ISM (a in Fig. 3), the labelling consisted of delineating the boundaries of consecutive objects with a polygon-type label in order to obtain an object mask, to extract the object from the image, and then to add it to the object pool. For the SSM (b in Fig. 3), areas representing only one class, e.g. chitin, were marked, and a polygon-type label was also obtained. These areas were then divided into smaller fragments using the Watershed algorithm, which were then added to the pools associated with the classes for semantic segmentation. Samples for larval segment classification for the LPM (c in Fig. 3) were obtained as follows. On an extracted live larva mask, two point labels were placed at the two ends in order to denote the head and abdomen end. The end segments were assigned to the head or abdomen end class depending on the annotator label, and the rest of the segments obtained the “normal” label (normal abdomen segments). The algorithm for dividing the larvae into segments was unsupervised, and is described in "Larvae Phenotyping" section.

#### Second stage of labelling

Obtaining an efficient and robust machine learning model requires iterative model development. This is not only related to retraining the models on enlarged datasets, but also to using previous models (so-called weak models) in order to achieve improved labelling. This makes it possible to quickly find the most difficult samples for inference, as well as to annotate them manually, which is the assumption of active learning^16^. Labelling samples for the instance segmentation model was very time-consuming. For this reason, an improved labelling process based on LabelTenebrioApp (d in Fig. 3) was proposed. At first, the annotator selected an image, together with previously obtained predictions, using the model with the best results so far. The annotation process itself involves a quick evaluation of the received predictions in terms of mask quality, and then predicted object class using a point-click technique. Objects with masks of good/acceptable quality and a true class label were added to the object pools without the need, as is the case with classical labelling, to draw a polygon. The most difficult cases that were not identified by the model were then manually labelled by the annotator.

### Pool-based 2D synthetic image generation

Developing machine learning models in the classical way, i.e., based on the completely manual annotation of random samples, is inefficient. This is especially noticeable for segmentation problems, when the label is a pixel annotation that requires a lot of effort from the annotator. For the undertaken issues, another problem is the high density of objects, their overlap, and their similarity, which increases uncertainty during labelling.

**Figure 4:**
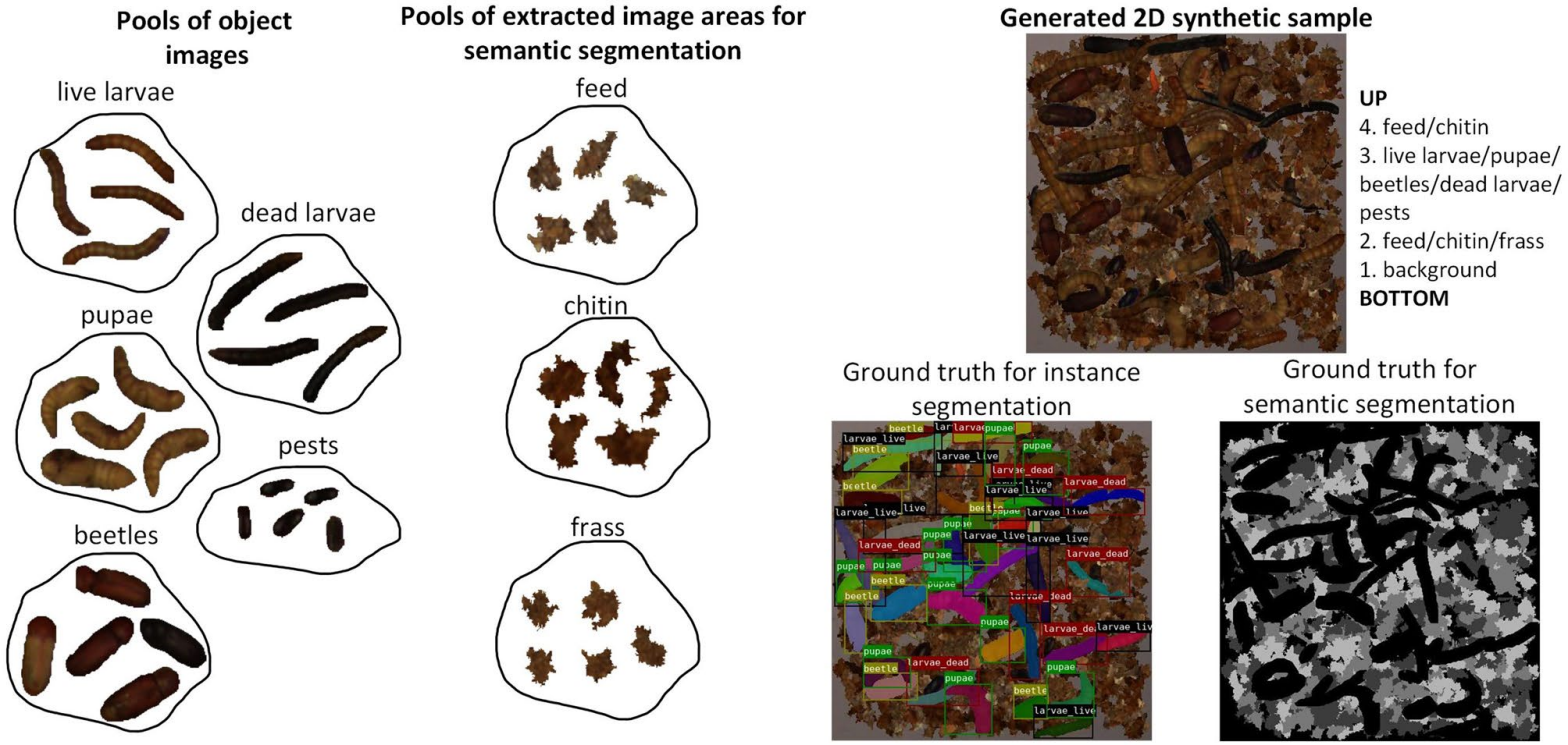
Pool-based 2D synthetic image generation method.

Considering these limitations, a semi-automatic method for generating 2D synthetic samples was proposed, which significantly reduced labelling time and uncertainty, and increased flexibility for iterative model development. The method is based on randomly placing elements in the form of images from the pools on the background image^17^. The extraction of items for the pools is described in "Labelling" section. In order to obtain a high similarity between the synthetic and real data, the elements were placed in a specific order. The first were fragments of feed, chitin, and frass, which are often found at the bottom of the box. Next, images of objects such as live larvae, pupae, beetles, dead larvae, and pests were placed in the image. Feed and chitin fragments were placed in the foreground, which is due to the fact that there could be possible feed residues after feeding, as well as moulting during larval growth. After the item placement procedure, a label was automatically generated in order to train the instance and semantic segmentation models without additional user supervision. A diagram of the proposed pool-based synthetic data generation method is shown in Fig. 4.

### Dataset

The labelling strategy described in "Labelling" section was applied to create the training datasets. A summary of the number of samples collected in this way is shown in Table 1.

**Table 1:** Number of objects in the training and test datasets.

Dataset type	No. of objects (ISM)					No. of polygons (SSM)			No. of segments (LPM)		
	Live larvae	Pupae	Beetles	Dead larvae	Pests	Feed	Chitin	Frass	Head	Normal	Abdomen end
Training	1026	1550	242	809	133	22	6	11	222	1879	225
Test	346	199	68	140	61	42	85	13	36	287	36

The numbers shown in Table 1 represent the number of objects for the ISM, the number of polygons for the SSM, and the number of segments for the LPM, respectively. The test datasets were prepared completely independently. In this case, the samples were labelled manually by an expert on varied images of boxes with Tenebrio molitor. The labelling process was performed similarly to the first labelling stage described in "Labelling" section except that the steps of preparing and adding items to the group of objects were omitted, e.g. areas were not divided into smaller fragments in the case of semantic segmentation. A summary of the number of test samples is given in Table 1. The numbers have analogous meanings to the training datasets.

### Instance segmentation with mask R-CNN

The Mask R-CNN^18^ model was used for the instance segmentation of objects from the classes: live larvae, pupae, beetles, dead larvae, pests. The Mask R-CNN complements the Faster R-CNN^19^, and has a part that is responsible for generating a mask for each detected object. The functioning of both the Mask R-CNN and Faster R-CNN is based on the determination of the region of interest by the Region Proposal Network (RPN) that is based on the feature map, which is the output of the convolutional neural network with the selected architecture. Once the region of interest sizes are unified, classification and boundary box regression using Fully Connected Layers is performed. The Mask R-CNN model additionally makes a mask prediction at this point. The loss minimised during training takes into account the accuracy of the described three model predictions, namely classification, bounding box regression, and mask extraction. In this study, ResNet-101^20^ was used as the feature map extractor, which is a common choice of researchers for similar problems^21^. The following hyperparameters were adopted for Mask-RCNN training: optimizer SGD, learning rate $2.5*10^{-4}$, iterations 10,000, weights mask_rcnn_R_101_FPN_3x. The research used the Mask R-CNN implementation from the Python library detectron2^22^.

### Semantic segmentation with U-net

The U-Net model^23^ was used for the semantic segmentation of the feed, chitin and frass areas from the images. U-Net has an autoencoder structure that consists of three main parts: an encoder, and a decoder with an identical structure and a bottleneck. The autoencoders encode information in the bottleneck and then decode it, resulting in the extraction of only the most important patterns from the data, as well as the reduction of noise. The dice loss minimised during training the model is based on comparing the model output with the ground truth. The values in the model output are scaled to the probabilities for the given classes using the softmax activation function. The final performance of the model is influenced by the choice of encoder and decoder architecture. For the issues undertaken, the EfficientNet-B0^24^ backbone pre-trained on ImageNet^25^ was chosen due to its efficiency and relatively small size. The following hyperparameters were adopted for U-Net training: optimizer Adam, learning rate $10^{-4}$, epochs 40. The study used the U-Net implementation from the Python library segmentation_models^26^.

### Larvae phenotyping

**Figure 5:**
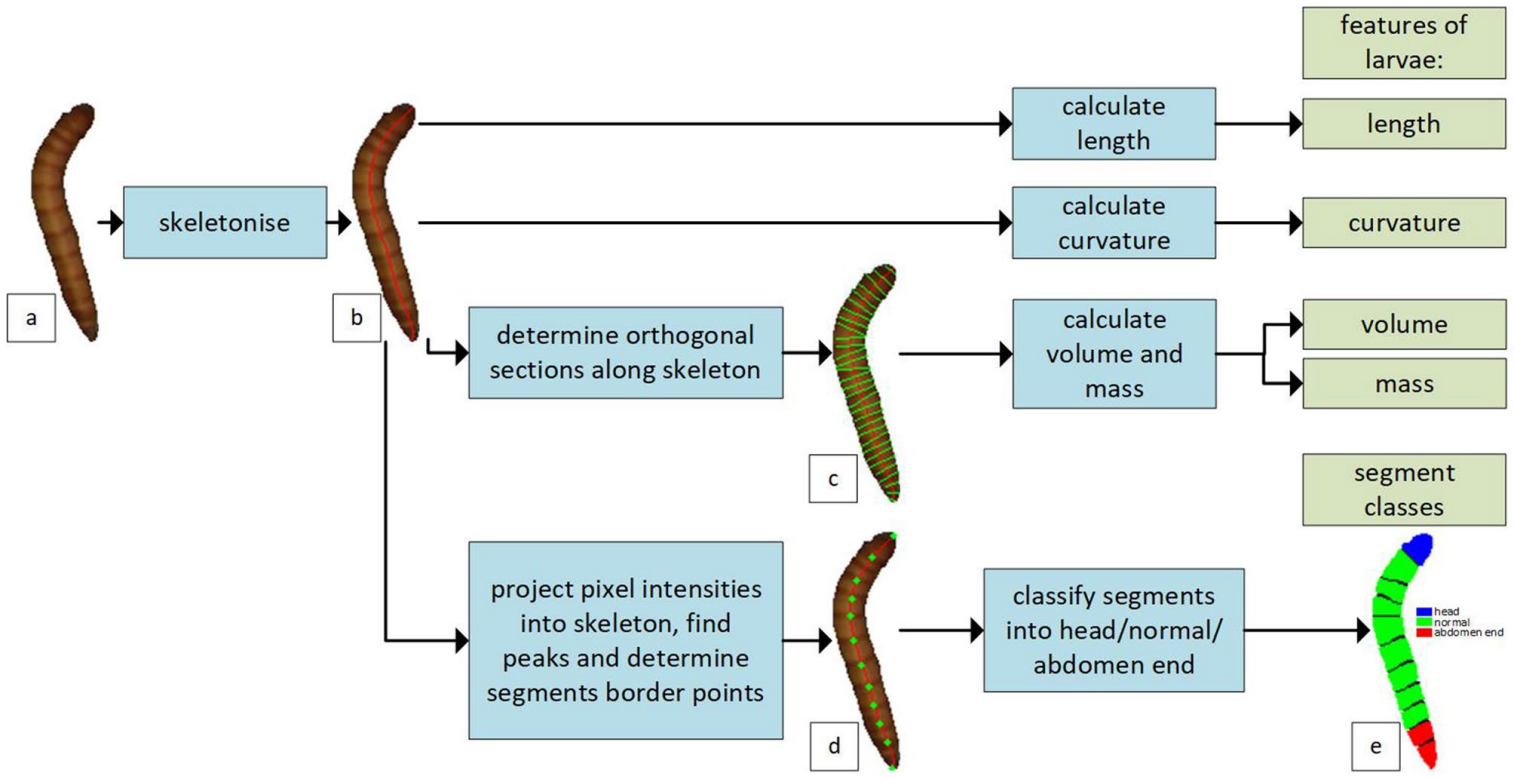
Larvae phenotyping schema: (**a**) raw image of the larva, (**b**) skeleton of the larva after skeletonisation, (**c**) orthogonal sections to the skeleton for calculating the volume of the larva, (**d**) segment boundary points marked on the skeleton, (**e**) segment classification.

The LPM allows the determination of the basic characteristics of individual larvae (length, curvature, mass, division into segments, and their classification ), and also the distribution of larval length for the whole population. This section describes the methods used in the LPM. The phenotyping scheme for single larvae is shown in Fig. 5.

#### Length calculation

Determining the length of larvae is not an obvious task due to the need to strictly define this dimension. In this research, the larval length was assumed to be the length of a curve going through the center of the larva along their largest dimension. To determine the described curve, the skeletonisation algorithm^27^ was used, along with a correction for boundary conditions, which involved drawing additional pixels at the ends of the curve while taking into account the local orientation of the skeleton and mask boundaries. The skeleton for an example larva is shown in image b in Fig. 5. By having the skeleton coordinates, the length was calculated from the following formula:

1 $$\begin{aligned} L=k\sum _{i=1}^{n-1}{l(S_{i+1}, S_{i})}=k\sum _{i=1}^{n-1}{\sqrt{(x_{i+1} - x_{i})^2+(y_{i+1} - y_{i})^2}} \end{aligned}$$

where $l(S_{i+1}, S_{i})$ - the Euclidean distance between consecutive points of the skeleton $S_{i+1}(x_{i+1}, y_{i+1})$ and $S_{i}(x_{i}, y_{i})$, *k* - the constant enabling the conversion from pixels to millimetres, *n* - the number of points in the skeleton.

#### Volume and mass calculation

When calculating the volume of larvae, it was assumed that it can be approximated by the total volume of a finite number of cylinders, the height $l_{i}$ of which is equal to the length of the defined skeleton section, and the diameter $d_{i}$ is equal to the length of the orthogonal section to the defined skeleton section that is contained within the binary mask, as shown in image c in Fig. 5. Once these quantities are determined, the value of the larvae volume can be calculated from the formula:

2 $$\begin{aligned} V=k^3c\sum ^{n-1}_{i=1}{\frac{\pi }{4}d_{i}^2l_{i}} \end{aligned}$$

where $l_{i}$ - the Euclidean distance between consecutive points of the skeleton, $d_{i}$ - the length of the section orthogonal to the considered section on the skeleton, *k* - the constant enabling the conversion from pixels to millimetres, *n* - the number of points in skeleton, *c* - the correction coefficient.

The use of correction factor *c* in the estimation of larval volume is due to the differences occurring between the real shape of the larvae and the ideal shape assumed in the study, which is especially affected by the flattening of the thorax near the head, and the lack of volume at the joints of the larval segments. Empirically, the value of the correction factor was determined to be $c=0.58$. The value of the constant *k* should always be determined individually during calibration using the length calibration standard (the value of the constant k depends on the resolution of the camera and the dimensions of the area of interest, e.g. box). A constant *k* equal to 0.153 [mm/pixel] was used in this study. For larvae mass calculations, the empirically determined density of mature Tenebrio molitor larvae equal to $\rho =1.31\pm0.25\frac{g}{cm^3}$ was used. The density was measured using a HumiPyc gas pycnometer.

#### Curvature calculation

Another calculated parameter was curvature. By knowing the coordinates of the skeleton, the value of curvature at a certain point *S*(*x*, *y*) can be calculated from the formula:

3 $$\begin{aligned} \kappa =\frac{|x'y''-y'x''|}{[(x')^2+(y')^2]^{\frac{3}{2}}} \end{aligned}$$

where *x* and *y* are coordinates of the skeleton points *S*(*x*, *y*), and $x', y', x'', y''$ are 1. and 2. order derivatives for a given coordinate.

The curvature of the larvae was calculated for the averaged coordinates of the skeleton points in the defined intervals with specific lengths. The final referenced curvature value is equal to the average curvature value at the specified points.

#### Division into segments and their classification

The larvae of Tenebrio molitor were composed of segments. The segments contained in the middle were similar and were characterised by a segment ending in the form of a dark band orthogonal to the skeleton. This pattern was used in the unsupervised division of the larva into segments.

First, the larvae images were converted from RGB to Lab colour space in order to use the L (lightness) channel. Afterwards, for each skeleton point, the average pixel intensity value was determined from the L-channel based on the closest larvae-forming points and a 255-L pixel intensity chart was generated along the determined skeleton. Peaks representing the boundary points of the segments were looked for in the prepared chart. Examples of boundary points representing peaks in the chart are shown in image d in Fig. 5. Based on the boundary points, segments were extracted and classified into head/normal/abdomen end.

To characterise the segments, 25 features were proposed: 12 intensity-related features (mean, skewness, kurtosis, entropy for each histogram R, G, B), 6 texture features (Haralick features^9^: contrast, dissimilarity, homogeneity. energy, correlation, ASM) and 7 shape features (Hu moments^28^). The synthetic minority over-sampling technique (SMOTE)^29^ was applied before classification due to unbalanced training data. Selection of the classification model was done by k-fold cross-validation using a training dataset. Finally, the best model was evaluated on an independent test dataset.

A summary of the number of samples in the training and test datasets for the segment classification problem is shown in Table 1. The models examined were logistic regression (LogReg), linear discriminant analysis (LDA)^30^, k nearest neighbours (KNN)^31^, and support vector machines (SVM)^10^. For the KNN and SVM models, hyperparameter optimisation was also performed by checking the number of neighbours for the KNN, and the type of kernel for the SVM. An example of segment classification is shown in image e in Fig. 5. For feature calculation and segment classification, the following Python libraries were used: Scipy^32^(intensity-related features), scikit-image^33^(Haralick features), OpenCV^34^(Hu moments) and scikit-learn^35^(ML models).

#### Length distribution estimation

The overlapping of larvae in the box results in the fact that only parts of some larvae can be seen in the image. If a larva is occluded, it should not be used to estimate the larval length distribution. To obtain a reliable histogram of larvae length in the box, only whole larvae from their head to abdomen end should be extracted from the image. For this purpose, the results of the segment classification described in "Larvae Phenotyping" section were used. A larva was accepted as proper if the last segments along its skeleton represented the head and abdomen end, respectively. Classification of the last segments as “normal” indicated overlapping.

### Evaluation

Evaluation was performed for the different tasks included in the monitoring system, namely: object detection (live larvae, pupae, beetles, dead larvae, pests), semantic segmentation (uneaten feed, chitin, frass), the estimation of larval length distribution, and the estimation of larval mass.

#### Object detection model evaluation

For object detection, the predictions and ground truths are in the form of rectangles called bounding boxes, which are described by 4 corner coordinates. Each prediction is also described by a confidence score value, which indicates the percentage of confidence in the prediction. Let us assume that for a given inference, a set of predictions $P=\{P_{1}, P_{2}, ..., P_{n}\}$ with confidence score values $C=\{C_{1}, C_{2}, ..., C_{n}\}$ were obtained and that the corresponding set of ground truths is $G=\{G_{1}, G_{2}, ..., G_{m}\}$. Moreover, each element from set P and G has the same label depending on the class under consideration. For each bounding box $G_{i}$, let us assign one bounding box $P_{j}$ for which: (1) the intersection over union $IoU\left( P_{j}, G_{i}\right) >=0.5$, and (2) $P_{j}$ has the highest $C_{j}$ among the predictions, which satisfies the 1st condition. The number of assignments between G and P is the number of True Positive (TP) predictions. Let us call the number of unassigned predictions from set P as False Positive (FP), and the number of unassigned ground truths from set G as False Negative (FN). From the determined TP, FP, and FN values, the precision and recall metrics can be calculated. Precision *PPV* (positive predictive value) defines the probability that a given prediction is correct, while recall *TPR* (true positive rate) defines the probability that a given ground truth object is detected. The formulas for precision and recall are as follows:

4 $$\begin{aligned}&precision=PPV=\frac{TP}{TP+FP} \end{aligned}$$

5 $$\begin{aligned}&recall=TPR=\frac{TP}{TP+FN} \end{aligned}$$

In order to characterise a model by a single value, a metric *F*1 is often used, which is the harmonic mean of precision and recall. The F1 metric can be calculated using the formula:

6 $$\begin{aligned} F1=\frac{2*PPV*TPR}{PPV + TPR} \end{aligned}$$

Since most of the predictions with a low confidence score are the source of FP errors, some of them that have a value below a certain threshold value $C_{thresh}$ are removed. On the other hand, too high a value of $C_{thresh}$ results in more FN errors. The optimal value of $C_{tresh}$ in such a case may be the value that maximises F1. The value of $F1_{opt}$ , based on the optimal operating point (threshold value $C_{opt}$), and the related metrics $PPV_{opt}$, $TPR_{opt}$ were used to evaluate object detection by the proposed models ($F1_{obj}$, $PPV_{obj}$, $TPR_{obj}$ ). To compare object detection models, it is also good practice to use metrics independent of $C_{thresh}$. The most commonly used metric that meets the threshold independence condition is average precision AP. AP is defined as the area under curve (AUC) precision x recall, as represented by the following formula^36^:

7 $$\begin{aligned} AP = \sum _{i=1}^{n-1} (r_{i+1} - r_{i})p_{interp}(r_{i+1}) \end{aligned}$$

where $p_{interp}(r_{i+1})= max$ $p({\tilde{r}})$, and ${\tilde{r}}:{\tilde{r}}>=r_{i+1}$, and *n* - the number of predictions.

The precision-recall chart is formed by the precision and recall values at different values of $C_{thresh}$. Before calculating the AUC, interpolation of the precision values for the chart points is performed, due to the lack of monotonicity (zigzag shape), with the raw precision x recall chart. For each recall value, the interpolated precision value must be greater than or equal to the precision value for the points with the greater recall value (all points to the right of the considered point), as described in the condition under formula 7. Due to the fact that a bounding box overlap threshold of 50% ($IoU=50\%$) was assumed, the average precision for detecting objects will be designated as $AP_{50}$.

#### Semantic segmentation model evaluation

Semantic segmentation is based on determining a label value for each pixel. Similar to object detection, the metrics $F1_{opt}$, $PPV_{opt}$, $TPR_{opt}$ for the optimal operating point can be used. The only difference is that individual pixels ($F1_{pix}$, $PPV_{pix}$, $TPR_{pix}$) are considered instead of objects. These three metrics were used in the study to evaluate the semantic segmentation models.

#### Larval segment classification model evaluation

The classification of larval segments was based on the prediction of the head/normal/abdomen end class for each detected segment. Similar to object detection and semantic segmentation, the metrics $F1_{seg}$, $PPV_{seg}$, $TPR_{seg}$ were used, with each incorrect or correct prediction being associated with one segment.

#### Larval length distribution estimation method evaluation

To validate the methods for estimating larval length distribution, independent test datasets were developed for three populations. Approximately 100 live larvae were selected from each population, and their images were registered. The length of the larvae was determined based on the collected images. A normalised histogram $h_{true}$ was then determined using the true larval lengths. The estimated normalised histogram $h_{est}$ was determined from the calculated larval lengths according to the method described in "Larvae Phenotyping" section and by using the filtering of occluded larvae, which was described in "Larvae Phenotyping" section The formula for the intersection of the histograms was used to determine the similarity of the histograms:

8 $$\begin{aligned} D(h_{true}, h_{est}) = \sum _{i=1}^{n} min(h^{true}_{i}, h^{est}_{i}) \end{aligned}$$

where $D(h_{true}, h_{est})$ - the intersection between two normalised histograms $h_{true}$ and $h_{est}$ ($\sum h^{true}_{i}=1$ and $\sum h^{est}_{i}=1$), and *n* - the number of bins.

Additionally, the means ($\overline{x}_{true}$, $\overline{x}_{est}$) and standard deviations ($\sigma _{true}$, $\sigma _{est}$) of the obtained distributions were compared.

#### Mass estimation method evaluation

Evaluation of the method for mass estimation consisted of comparing the true and estimated mass of the larvae in the box. For this purpose, the following experiment was conducted for three different populations. 10 grams of live larvae were added to the box. The total mass of the larvae in the box then ranged from 0 to 100 grams. After each procedure of putting larvae into the box, an image was registered. Each such image was then input to the developed model for instance segmentation to determine the masks for the live larvae. The mass of all the larvae in the box was estimated based on the procedure described in “Volume and mass calculation” section.

The squared Pearson correlation coefficient $R^{2}$ between the true mass $m_{true}$ values and the estimated mass $m_{est}$ values was used as a quantitative indicator of the estimation quality:

9 $$\begin{aligned} R^2 = r_{m_{true},m_{est}}^2 = \frac{cov^2(m_{true}, m_{est})}{\sigma _{m_{true}}^2\sigma _{m_{est}}^2} \end{aligned}$$

where $\sigma _{m_{true}}$, $\sigma _{m_{est}}$ - standard deviations of $m_{true}$ and $m_{est}$, and $cov(m_{true}, m_{est})$ - the covariance of $m_{true}$ and $m_{est}$.

Due to significant overlapping of larvae when there are high numbers of larvae in the box, the $R^{2}$ ratio was determined for the initial values of the true larval mass in the box (from 0 to 40 grams). The slope of the line $a_{0-40}$ in the considered interval was used as an additional parameter of the quality of the mass estimation.

## Results and discussion

This section contains the evaluation results of the different machine learning models, and also the methods used in the proposed modules: ISM, SSM and LPM. The evaluation was performed on test datasets that are independent of the training datasets. Samples for the test datasets were labelled on real images of the boxes with Tenebrio molitor. A summary of the number of samples in the training and test datasets is presented in Table 1. An explanation of the metrics used can be found in "Evaluation" section.

**Figure 6:**
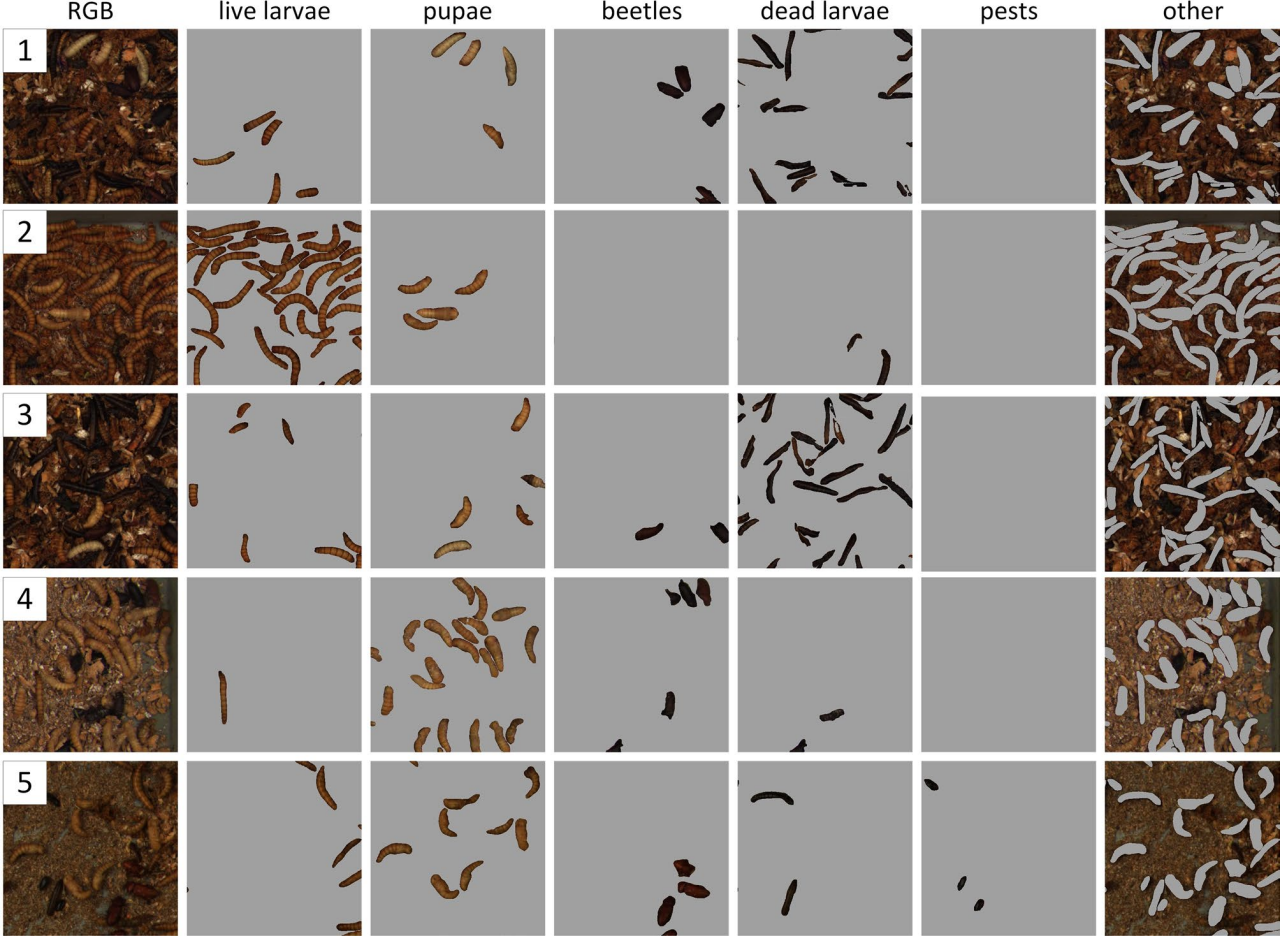
Results of the instance segmentation for live larvae, pupae, beetles, dead larvae, and pests for the sample data.

The detection results of the Tenebrio molitor growth stages (live larvae, pupae, beetles) and anomalies (dead larvae, pests) for the Mask R-CNN model with the ResNet-101 backbone are presented in Table 2 and in Fig. 6.

**Table 2:** Results of the detection growth stages of Tenebrio molitor (live larvae, pupae, beetles) and anomalies (dead larvae, pests) for the Mask R-CNN with the ResNet-101 backbone.

	Type	$AP_{50}$	$F1_{obj}$	$PPV_{obj}$	$TPR_{obj}$
Live larvae (Tenebrio molitor)	Growth-stage	0.915	0.905	0.927	0.884
Pupae (Tenebrio molitor)		0.900	0.893	0.929	0.859
Beetles (Tenebrio molitor)		0.934	0.930	0.984	0.882
Dead larvae (Tenebrio molitor)	Anomaly	0.814	0.858	0.898	0.821
Pests (Alphitobius diaperinus)		0.777	0.835	0.889	0.787

The proposed instance segmentation model detected growth stages ($F1_{obj} > 0.89$) and anomalies ($F1_{obj} > 0.83$) very efficiently. The density of objects, and their overlapping with each other, did not destructively affect the model’s performance—both whole objects and their fragments were detected well (samples 2–4 in Fig. 6 for a high density of live larvae, dead larvae, and pupae, respectively). The robustness of the model to dense scenes is due to the proposed 2D synthetic data generation method described in "Pool-based 2D synthetic image generation" section, where dense scenes with different types of objects that overlap with accurate pixel annotation were simulated. Based on the values of the metrics in Table 2, it can be seen that $PPV_{obj} > TPR_{obj}$ for all the considered classes. This results in a fewer number of committed FP errors, which comes at the expense of more undetected objects. This choice of operating point is appropriate for anomaly detection, as it reduces the number of possible interventions by the farmer. However, anomalies will mostly be detected anyway due to the presence of more than one object from the anomaly class in the box (samples 3 and 5 in Fig. 6 for dead larvae and pests). Some problems, due to the high cost of non-detection, e.g., detecting the first beetle in the context of breeding interruption, require the operating point to be moved to higher recall values. The few errors made during inference were mainly: (1) misclassification of object fragments between the live larvae and pupae classes (samples 2–3 in Fig. 6), (2) misclassification of object fragments between the dead larvae and beetles classes (sample 4 in Fig. 6), and (3) undetected object fragments in dense scenes (samples 2–4 in Fig. 6 for the live larvae, dead larvae, and pupae classes, respectively). However, these errors do not affect the usefulness of the proposed ISM.

Mask R-CNN, as a representative of an instance segmentation model for detecting different Tenebrio molitor growth stages and anomalies, was chosen for this study due to: (1) the ability to further phenotype the detected objects based on their binary masks, (2) the need to extract the instances pixel-wise in order to add them to the object pool (described in "Pool-based 2D synthetic image generation" section), and (3) the simplicity to analyse the prediction and to draw conclusions to further improve the models. Although Mask R-CNN works well in the model development stage, the rationale for its use in the final model should be considered by taking into account the required functionality of the system. If only counting objects from defined classes is required, an object detection model such as YOLO^37^ (with significantly less inference time than Mask R-CNN) may be a better solution. As part of the study, the Mask R-CNN with the backbone ResNet-101 model and the YOLOv5x^38^ model were also compared in terms of $AP_{50}$ and inference time for the tile, as shown in Table 3. The GeForce RTX 2060 SUPER 8GB (GPU) and AMD Ryzen 7 1700 3GHz (CPU) were used to measure the inference time for the Mask R-CNN and YOLO models.

**Table 3:** Comparison of object detection $AP_{50}$ and inference time for the Mask R-CNN and YOLO models.

Model	$AP_{50}$					Inference time/tile
	Live larvae	Pupae	Beetles	Dead larvae	Pests	
Mask R-CNN (ResNet-101)	0.915	0.900	0.934	0.814	0.777	120 ms
YOLOv5x	0.894	0.893	0.921	0.700	0.738	40 ms

The $AP_{50}$ values in Table 3 for all the considered classes (except the dead larvae), when comparing the Mask R-CNN (ResNet-101) and YOLOv5x models, decreased slightly. Moreover, the inference time decreased about three times. Taking into account that the RGB image of the whole box (example shown in Fig. 1) was split into 192 standard size tiles before inference, and the fact that the total inference time is notable, use of the YOLOv5x model in the final monitoring system can be seen to be appropriate.

**Figure 7:**
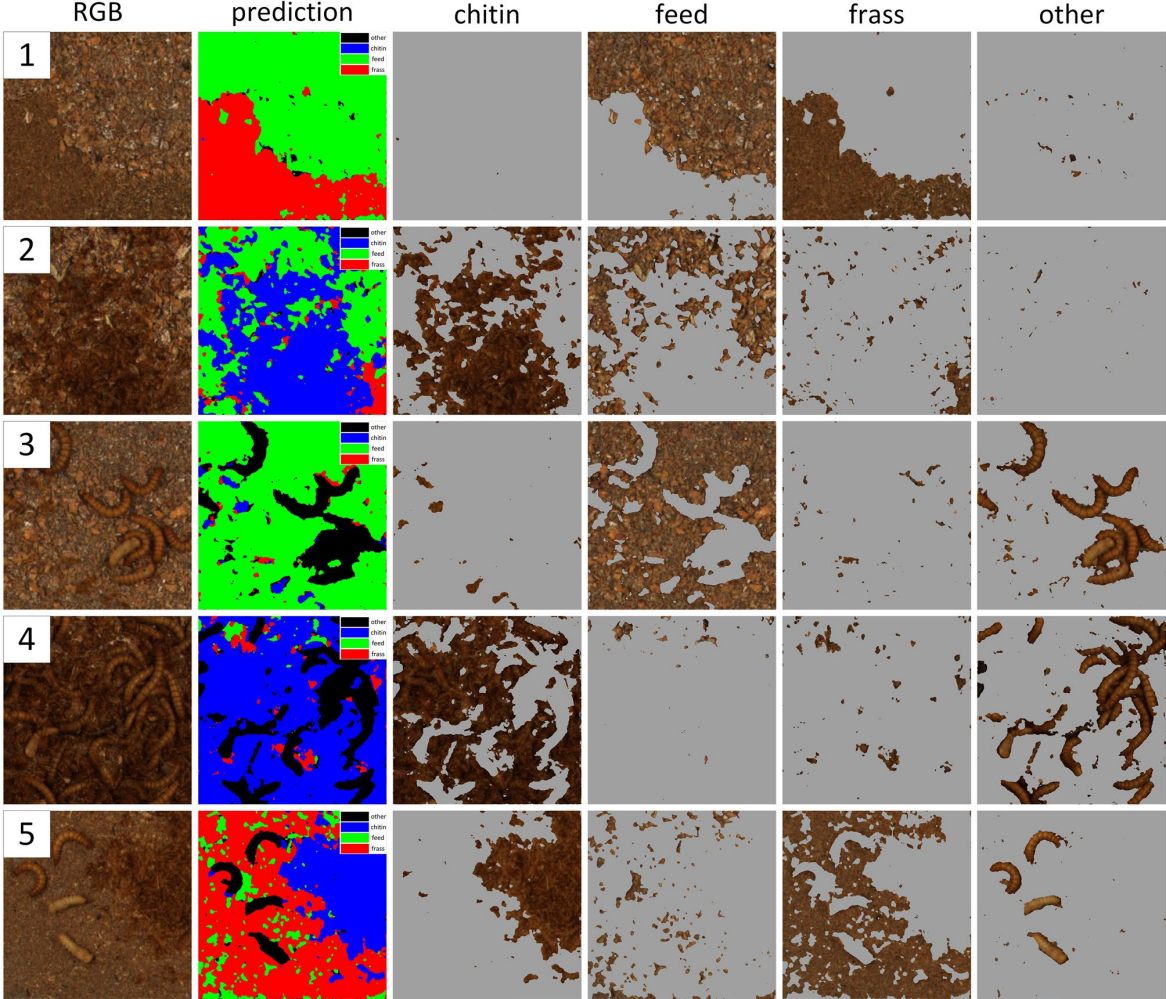
Results of the semantic segmentation of the feed, chitin and frass for the sample data.

The semantic segmentation results for the U-Net model with the EfficientNet-B0 backbone are presented in Table 4 and in Fig. 7.

**Table 4:** Results of the semantic segmentation for the feed, chitin and frass for the U-Net with the EfficientNet-B0 backbone.

	$F1_{pix}$	$PPV_{pix}$	$TPR_{pix}$
Feed	0.971	0.969	0.973
Chitin	0.947	0.918	0.977
Frass	0.953	0.963	0.943

In the case of the semantic segmentation, the achieved values of $F1_{pix} > 0.94$ for the feed, chitin and frass classes demonstrate the ability of the model to efficiently segment the regions that represent the defined classes. The SSM was able to cope with both the segmentation of the larger regions of a class, as well as smaller regions, e.g., one feed flake, one chitin moult, as can be observed on samples 1–5 in Fig. 7. The most common inference errors for the SSM were: (1) mistakes between the feed and frass classes (samples 1 and 5 in Fig. 7) for areas without cereal grains, (2) chitin segmentation at the end segments (head, abdomen end) of the live larvae (sample 5 in Fig. 7), and (3) mistakes in the small areas between objects e.g. larvae (samples 3 and 4 in Fig. 7). For the SSM, it is important to note the very fast development process of the model. The proposed labelling method described in "Labelling" section (splitting a larger annotated area into smaller ones and adding them to a pool), together with the generation of synthetic samples described in "Pool-based 2D synthetic image generation" section, enabled a significant reduction in the model’s development time. Moreover, the model maintained a comparable diversity of the samples and an increase in label veracity when compared to the classical labelling of the samples for the semantic segmentation.

**Figure 8:**
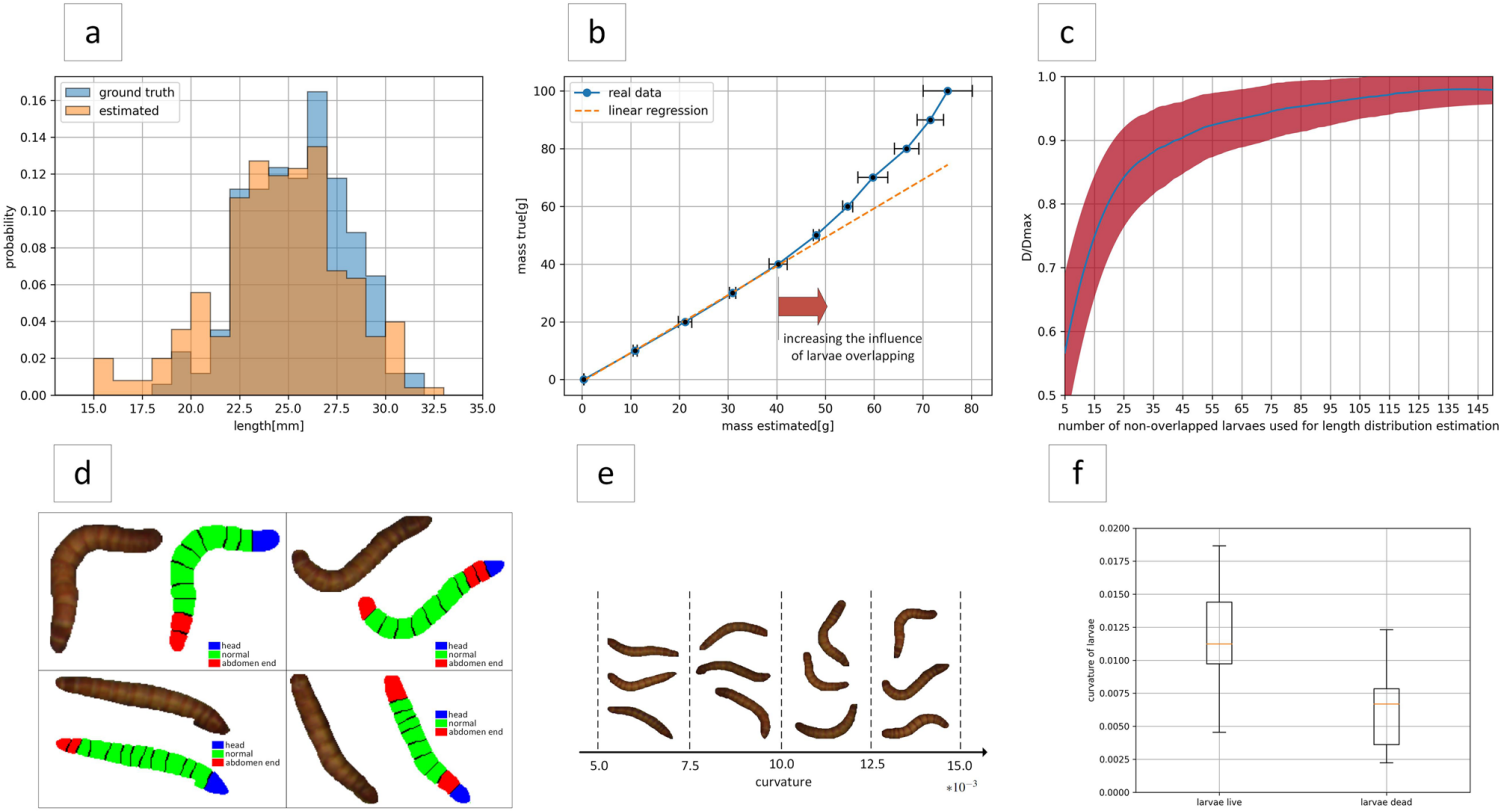
Charts related to larval phenotyping: (**a**) comparison of true and estimated larval length distribution for a selected population, (**b**) estimation of larvae mass based on averaged samples, (**c**) chart of the normalised intersection of histograms as a function of the number of non-overlapping larvae used to estimate the larval length distribution, (**d**) segment classification for example larvae using the best proposed model, (**e**) examples of larvae for specific ranges of curvature values, (**f**) comparison of curvature for live and dead larvae.

The classification of larval segments was one of the components of the LPM. Evaluation results on the test dataset for larval segment classification for the top three models (LDA, SVM, LogReg) were shown in Table 5. In Fig. 8, selected errors made by the classifiers can be analysed. Most of the incorrect predictions are related to the classification of segments located before the end segments. By analyzing the characteristics of these mistakes, it is easy to eliminate them by treating duplicate head or abdomen end predictions of neighbouring segments as one prediction. In Table 5, it can be seen that the proposed prediction processing method taking segment location into account significantly increased the examined metrics for all shown models. Considering the averaged metrics after prediction processing, the best model for segment classification was LDA, which was characterized by $F1_{score} > 0.75$ for each considered class: head/normal/abdomen end. Its usefulness in filtering whole larvae from fragments was confirmed by the high intersection values of the larval length histograms ($D(h_{true}, h_{est}) > 0.8$).

**Table 5:** Results of the classification of the segments of the live larvae into head, normal and abdomen end.

Segment type	Model	$TPR_{seg}$		$PPV_{seg}$		$F1_{seg}$	
		Raw predictions	Processed predictions	Raw predictions	Processed predictions	Raw predictions	Processed predictions
Normal	LDA	0.895	0.983	0.988	0.989	0.939	0.986
	LogReg	0.930	0.990	0.996	0.996	0.962	0.993
	SVM	0.920	0.986	1.000	1.000	0.958	0.993
Head	LDA	0.833	0.833	0.600	0.769	0.698	0.800
	LogReg	0.750	0.750	0.692	0.794	0.720	0.771
	SVM	0.750	0.750	0.692	0.794	0.720	0.771
Abdomen end	LDA	0.750	0.750	0.551	0.771	0.635	0.760
	LogReg	0.806	0.806	0.558	0.725	0.659	0.763
	SVM	0.833	0.833	0.536	0.714	0.652	0.769
All (average metrics)	LDA	0.826	0.855	0.713	0.843	0.757	0.849
	LogReg	0.829	0.849	0.749	0.838	0.780	0.842
	SVM	0.834	0.856	0.743	0.836	0.777	0.844

**Table 6:** Results of estimating the larval length distribution and mass for the three study populations.

Population id	$\overline{x}_{est} [mm]$	$\sigma _{est} [mm]$	$\overline{x}_{true} [mm]$	$\sigma _{true} [mm]$	$D(h_{true}, h_{est})$	$R^2_{0-40}$	$a_{0-40}$
1	27.4	3.1	27.8	3.3	0.806	0.998	1.02
2	24.6	3.3	25.5	2.6	0.842	0.999	0.95
3	26.5	2.3	26.2	1.9	0.874	0.999	1.04

The results obtained for the estimation of the larvae length distribution (Table 6 and Chart a in Fig. 8) prove that the developed method for determining these quantities is efficient. The high histogram similarity values obtained ($D(h_{true}, h_{est}) > 0.8$) exceed the requirements for a monitoring system. Taking into account that the number of visible larvae in a box can reach 1000, it is necessary to consider the validity of phenotyping all visible larvae, which is computationally expensive. To this purpose, a chart was prepared of the normalised intersection of histograms (relative to the maximum intersection value obtained when using all larvae for estimation) as a function of the number of non-overlapping larvae used to estimate the larval length distribution, which is shown in Chart c in Fig. 8. This chart shows that adding more larvae (above 45 individuals) to the estimation no longer contributes significantly to the histogram intersection, while 45 individuals allows a value of about 0.9 of the maximum histogram intersection to be achieved, which is definitely an acceptable compromise. From Chart a in Fig. 8, it can be observed that histogram mismatches occur mainly in the tails of the distributions: (1) the underestimation of the number of longer larvae (right tail) results from the higher probability of such larvae being occluded under breeding conditions, while (2) the overestimation of the number of shorter larvae (left tail) results from the few errors during segment classification and the fact that occluded larvae are taken for estimation. The occurrence of the mentioned problems does not negate the usefulness of the proposed method for estimating the larval length distribution for larval growth monitoring during breeding.

The results for the larvae mass estimation problem in Table 6, and Chart b in Fig. 8, confirm that the proposed method for estimating the volume and mass of larvae is appropriate, as indicated by the values of the metrics for the range from 0 to 40 grams ($R_{0-40}^2>0. 99$ and $|1-a_{0-40}|<6\%$). However, its applicability under breeding conditions with high larval overlap is limited. This method can mainly be seen to have potential in experiments with relatively small numbers of larvae, e.g. testing new types of feed in laboratory breeding studies, when it allows for the non-invasive determination of larval weight gains. A solution for an effective vision-based determination of larval weight in the box under real breeding conditions may be a hybrid approach. Using the knowledge of the larval length distribution, larval growth stage, and approximate number of individuals per box (at the beginning of breeding it is similar for all boxes), a model can be developed to also estimate the mass of unseen larvae. Verification of this idea is the next direction of our research.

The proposed curvature parameter may be one of the indicators of larvae health, so it was included in the LPM. Images of larvae with different curvature values are shown in Chart e in Fig. 8. The stiffening phenomenon (reduction in the value of the curvature parameter) was observed when the larvae die, as shown in Box chart f in Fig. 8, which compares the curvature distribution for live and dead larvae. The stiffening phenomenon could also be observed in the case of larvae/pupae transformation. Preliminary studies show the potential of the curvature parameter for larval phenotyping, but investigating its usefulness is a topic for further studies based on long-term observations.

The obtained evaluation results of the developed modules give attitudes to believe that the proposed system can be used in a real scenario. The study proposed four versions of the system usage depending on the needs of the user (researcher or breeder), as shown in Table 7. The first (full version) of the system assumes accurate image analysis of the entire box (192 tiles in total, which includes additional tiles for reducing edge effect related to the difficulty of detecting objects on the edges of a tile) using Mask-RCNN for ISM and U-Net for SSM. Phenotyping in the first version includes computation of all proposed features for selected 50 larvae from the box. The second version of the system includes the analysis of 25 percent of the box area (without reducing edge effects) using the same models as in the first version. Phenotyping in the second version includes computation of the proposed geometric features, i.e. length, volume, and curvature, without division into segments, and their classification. The third version limits the phenotyping of larvae only to the basic parameter of the binary mask area and the rest of the assumptions are the same as in the second version. In the fourth version, larval phenotyping is dispensed with, allowing the model to be changed from Mask R-CNN to YOLOv5x in the ISM module. The presented versions of the system represent a trade-off between the amount of population information obtained and the inference time (the number of boxes that can be analyzed per day) and the rationale of their use depends on the needs of the user. Undoubtedly, options 3 and 4 can be considered for use in monitoring large-scale edible insect breeding (30,000 and 54,000 analyzed boxes per day). The use of options 1 and 2 should be seen in monitoring smaller farms and for laboratory breeding studies. It should also be noted that larval phenotyping is the bottleneck of the whole system and future work should focus on increasing the efficiency of the LPM module.

**Table 7:** Computational burden analysis for four versions of the proposed system.

x	% of box area	ISM		SSM		LPM		Total time per box	No. of boxes per day
		Model	Time per box	Model	Time per box	Parameters	Time per box		
1	100 (192 tiles)	Mask R-CNN	30.6 s	U-Net	8.7 s	Proposed all	186.4 s	225.7 s	380
2	25 (12 tiles)	Mask R-CNN	2.0 s	U-Net	0.5 s	Proposed geometric (length, volume, curvature)	52.4 s	54.9 s	1570
3	25 (12 tiles)	Mask R-CNN	2.0 s	U-Net	0.5 s	Basic (area of binary mask)	0.4 s	2.9 s	30,000
4	25 (12 tiles)	YOLOv5x	1.1 s	U-Net	0.5 s	–	–	1.6 s	54,000

The application of our system in a breeding environment requires the preparation of appropriate hardware and software architecture. Potential users of the system should pay attention to the following recommendations: (1) computer with GPU (or external server with GPU) enabling fast prediction by proposed ML models, (2) periodic automated boxes inspection (frequency depending on requirements, initially once every day or two days may be assumed), (3) database containing calculated features for each analyzed box, (4) identification of individual boxes with edible insects (e.g. RFID), (5) application (Web, mobile) for the farmer, including reporting of anomalies and with the possibility to view historical data (changes of characteristics over time for a specific box), (6) additional system to control and optimize the breeding process based on current data.

## Conclusions

The developed multipurpose monitoring system for the breeding of Tenebrio molitor based on three modules (ISM, SSM and LPM) has great potential for the observation of edible insects in both laboratory breeding studies and real breeding conditions. The proposed method for developing multiclass instance and semantic segmentation models based on synthetic image generation and object pools significantly reduced the time of the iterative improvement of machine learning models, while also increasing the robustness of the models to problems such as dense scenes and the detection of minority class objects. The described method for estimating the length distribution of larvae in a box enables effective supervision of larval growth during breeding, even when most larvae are invisible. The developed larval mass estimation methods can be successfully applied to feeding experiments for the non-invasive assessment of mass gains. Future work will include: (1) the improvement of the synthetic image generation process and the quality of generated images, (2) the improvement in efficiency (inference time reduction) for the larvae phenotyping module, (3) the extension of the larvae phenotyping module to include more features for the growth stages of Tenebrio molitor (also for pupae and beetles), (4) the development of a module to determine larval activity using temporal data based on optical flow, (5) the long-term observation of Tenebrio molitor in order to characterise the change in states (live larvae to dead larvae, live larvae to pupae) and behavioural patterns, (6) the association of larval features with symptoms of disease or poor condition, and (7) re-identification for individual larvae and beetles.

## Data availability

The samples used to develop the ISM, SSM and LPM modules and the generated sample synthetic data are available from the corresponding author on reasonable request.
